# Efficacy of the Chinese herbal medicine Jintiange capsules in the postoperative treatment of osteoporotic vertebral compression fractures: a systematic review and meta-analysis

**DOI:** 10.3389/fmed.2023.1289818

**Published:** 2023-12-15

**Authors:** Yongsheng Fu, Weiguo Wang, Minghua Zhao, Jianpeng Zhao, Mingyue Tan

**Affiliations:** ^1^First Clinical Medical College, Shandong University of Traditional Chinese Medicine, Jinan, China; ^2^Affiliated Hospital of Shandong University of Traditional Chinese Medicine, Jinan, China

**Keywords:** Chinese herbal medicine, Jintiange capsules, osteoporosis, osteoporotic vertebral compression fractures, systematic review and meta-analysis

## Abstract

**Background:**

In traditional Chinese medicine, Jintiange capsules are frequently used to treat metabolic bone diseases and strengthen bones and tendons. The main component of Jintiange capsules is bionic tiger bone powder. However, the active ingredients and proteins are derived from other animal bones, with chemical profiles similar to that of natural tiger bone. This study aimed to explore the efficacy of Jintiange capsules, a Chinese herbal medicine, in the postoperative treatment of osteoporotic vertebral compression fractures (OVCFs).

**Methods:**

In this systematic review, literature was retrieved using PubMed, the Cochrane Library, the Chinese National Knowledge Infrastructure, the Web of Science, the Wanfang Database, the Chinese Biomedical Literature Database, and the Chinese VIP Database from inception to July 2023. The primary outcome measures were the bone mineral density (BMD) and effective rate. The secondary outcome measures were the visual analog pain score (VAS), Oswestry disability index (ODI), Cobb’s angle, serum osteocalcin, serum alkaline phosphatase, and adverse events. RevMan 5.4 and STATA 17.0 software were used for data analysis.

**Results:**

We enrolled randomized controlled trials (RCTs) focusing on 1,642 patients in the meta-analysis. The meta-analysis illustrated that Jintiange capsules significantly increased the BMD of the lumbar spine (*p* < 0.00001), femoral neck (*p* = 0.0005), and whole body (*p* = 0.01). The subgroup analysis of Jintiange capsules combination therapy showed that the BMD of the lumbar spine and whole body was significantly improved with Jintiange capsules (*p* < 0.00001). The test for the overall effect showed that Jintiange capsules had a significantly higher effective rate than the control groups (*p* = 0.003). Additionally, the overall effect test showed that Jintiange capsules decreased the VAS and ODI (*p* < 0.00001) and Cobb’s angle (*p* = 0.02), and improved serum OC and ALP (*p* < 0.00001) compared with the controls. Furthermore, the pooled analysis of adverse reactions showed no serious impacts on the treatment of OVCFs.

**Conclusion:**

Jintiange capsules demonstrate high safety and efficacy in the treatment of OVCFs, including increasing BMD, the lift effect rate, serum OC levels, and pain relief, decreasing the ODI, serum ALP levels, and adverse events, and improving Cobb’s angle. Additional research is required to validate the efficacy of Jintiange capsules for the postoperative treatment of OVCFs.

**Systematic review registration**: https://www.crd.york.ac.uk/PROSPERO.

## 1 Introduction

Osteoporosis (OP) is a systemic disease characterized by low bone mass, decreased bone strength, increased skeletal fragility, and bone tissue destruction (1). Osteoporosis affects approximately 10 million people aged over 50 years in the United States and 34.65% of those over 50 years of age in China (2). Among older adults, osteoporotic vertebral compression fractures (OVCFs) are one of the most severe manifestations of osteoporosis (3). The clinical symptoms of OVCFs, including severe kyphosis, lower back pain, and frequent relapse, impact the physical and mental health, quality of life, and life expectancy of patients with OVCFs (4). The most common non-surgical treatment of OVCFs is bed rest, analgesics, and estrogen replacement therapy (5). Although beneficial, these have several side effects that prevent pain relief and progression over the long term (6). Although percutaneous vertebroplasty and percutaneous kyphoplasty (PVP/PKP) are frequently used to treat OVCFs, they do not relieve osteoporosis-related pain or prevent adjacent vertebral fractures (7). Therefore, developing alternative medications with fewer adverse effects and novel therapeutic techniques for postoperative OVCF treatment is essential and feasible.

Currently, bisphosphonates, calcium agents, and estrogens are widely used to prevent and treat OVCFs. Although numerous anti-OVCF medications with diverse pharmacological properties are available, the targeted therapeutic effect is not attained in significant numbers of individuals with OVCFs (8). Some studies have shown that bisphosphonates can selectively adhere to and remain within the bone and promote the apoptosis of osteoclasts, but the long-term use of the medication can lead to gastrointestinal side effects and induce bone microdamage accumulation and atypical insufficiency fractures in the skeletal system (9). Calcium has been reported to increase the risk of myocardial infarction in the long term because OVCFs require long-term treatment, in which the harm and benefits of medications are unavoidable and need to be well balanced (10).

Traditional Chinese medicine has long been a treatment for OP, tonifying the kidneys and strengthening the bones in accordance with Chinese medicine treatment principles (11). For the treatment of OP, natural Chinese herbal medicine possesses distinctive advantages that improve bone quality and biomechanical properties and influence the fate of bone cells during bone remodeling (12). Jintiange capsules are a common Chinese medicine, the primary ingredient of which is artificial tiger bone powder (13). Modern pharmacological studies have demonstrated that tiger bone is abundant in collagen, amino acids, and minerals and can play important anti-inflammatory and analgesic roles as well as strengthening tendons and bones (14). Existing evidence shows that natural tiger bone can reduce fracture risk and relieve pain in OVCFs (15). However, the use of natural tiger bone is prohibited because the tiger is a protected animal in China. To meet the medical demand, artificial tiger bone powder has been developed to substitute natural tiger bone, and its structure closely resembles that of natural tiger bone (16, 17). Several clinical studies have indicated that multiple organic constituents of Jintiange capsules, such as amino acids, calcium, magnesium, phosphorus, iron, copper, manganese, and zinc, can improve bone formation, inhibit bone absorption, and accelerate the metabolism to absorb calcium for the postoperative treatment of OVCFs (18, 19). Although Jintiange capsules have been regarded as an effective Chinese patent medicine for patients with OVCFs in China and are recommended in Chinese OP treatment guidelines, there is currently a lack of high-quality evidence regarding the efficacy of Jintiange capsules in the treatment of OVCFs (20). Therefore, it is imperative to conduct this Systematic Review and meta-analysis to evaluate the efficacy of Jintiange capsules in the postoperative management of OVCFs.

## 2 Materials and methods

This study strictly followed the Preferred Reporting Items for Systematic Reviews and Meta-Analyses (PRISMA) statement (21). The ethical review was unnecessary because the data from the articles included in this meta-analysis were publicly available. The study was registered on PROSPERO (CRD42023456068).

### 2.1 Search strategy

We conducted an electronic search of the following seven authoritative literature databases: PubMed, the Cochrane Library, the Chinese National Knowledge Infrastructure (CNKI), the Web of Science, the Wanfang Database, the Chinese Biomedical Literature Database, and the Chinese VIP Database from their inception until July 2023. Furthermore, each database employed a combination of MeSH terms and free words for comprehensive study searches, and the language of the published literature was unrestricted. The relevant keywords for Jintiange were “Jintiange capsule” OR “artificial tiger bone” OR “Jintiange” OR “bionic tiger bone.” We retrieved keywords of OP, including “osteoporosis” OR “postmenopausal osteoporosis” OR “bone mass” OR “bone loss” OR “bone mineral density.” The keywords for OVCFs were “compression fractures” OR “lumbar compression fracture” OR “thoracic vertebral compression fractures” OR “osteoporosis vertebral compression fractures” OR “OVCFs.”

### 2.2 Eligibility criteria

We only included RCTs that compared Jintiange capsules with conventional western therapies and placebos for the postoperative treatment of OVCFs. The intervention group used Jintiange alone or with other conventional western therapies. The control group received only conventional western therapies or a placebo. Participants were diagnosed with OVCFs regardless of age, gender, dosage, duration, or disease course. Primary outcomes included bone mineral density (BMD) and effective rate; secondary outcomes included visual scale (VAS), the Oswestry Disability Index (ODI), Cobb’s angle, serum osteocalcin (OC), and adverse events. Additionally, all outcome indicators had to specify the results of the pooled data.

### 2.3 Exclusion criteria

The exclusion criteria included the following: (1) non-randomized controlled trials; (2) participants that were not diagnosed with OVCFs; (3) RCTs with participants who did not receive postoperative treatment; (4) studies that did not use Jintiange capsules or any other Chinese herbals or Traditional Chinese Medicine therapies; and (5) meeting abstracts, reviews, duplicate publications, animal experiments, and insufficient clinical data.

### 2.4 Data extraction

Two researchers extracted and collated the basic information of the final studies based on inclusion and exclusion criteria. Additionally, a third researcher examined the results independently. For this meta-analysis, author, publication year, study type, sample size, age of participants, operation method, number of study centers, follow-up time, intervening measures, and course of treatment were required as general information about the study. Disagreements between the two researchers about the pooled data were determined and resolved by consulting with a third researcher.

### 2.5 Quality assessment of the included studies

Two independent authors evaluated the risk of bias for each trial study using the Cochrane systematic reviews. This evaluation tool has seven items: random sequence generation (selection bias), allocation concealment (selection bias), blinding of participants and personnel (performance bias), blinding of outcome assessment (detection bias), incomplete outcome data (attrition bias), selective reporting (reporting bias), and other bias. Each domain can be judged as low bias risk, high bias risk, and unclear bias risk in the literature (22, 23).

### 2.6 Statistical analysis

All the enrolled studies were conducted on the Review Manager Software (RevMan5.4), and the STATA software (STATA Software Version 17.0) was used to evaluate publication bias and execute sensitivity analysis. The continuous outcome variables were presented as mean difference (MD) with a 95% confidence interval (95% CI), and we measured the same outcomes using standardized mean differences (SMD) of 95% CIs. We extracted the number of positive events based on binary outcome variables and calculated the risk ratio (RR) with 95% confidence intervals for both categories. The heterogeneity of the trial was assessed using *I*^2^ statistics, among which *p* < 0.05 or *I*^2^ > 50% was evaluated to have high heterogeneity, and we used a random-effects model. Each article that contributed to heterogeneity was excluded from the sensitivity analysis; otherwise, the fixed-effects model was used when the heterogeneity between the enrolled studies was small (*p* ≥ 0.05 or *I*^2^ ≤ 50%). Furthermore, subgroup analysis was applied to the trial based on Jintiange capsules, separated into Jintiange combined intervention subgroup and Jintiange alone intervention subgroup. A funnel plot was constructed to evaluate potential publication bias, which was analyzed using Egger’s test.

## 3 Results

### 3.1 Screening results of the studies

**Figure 1:**
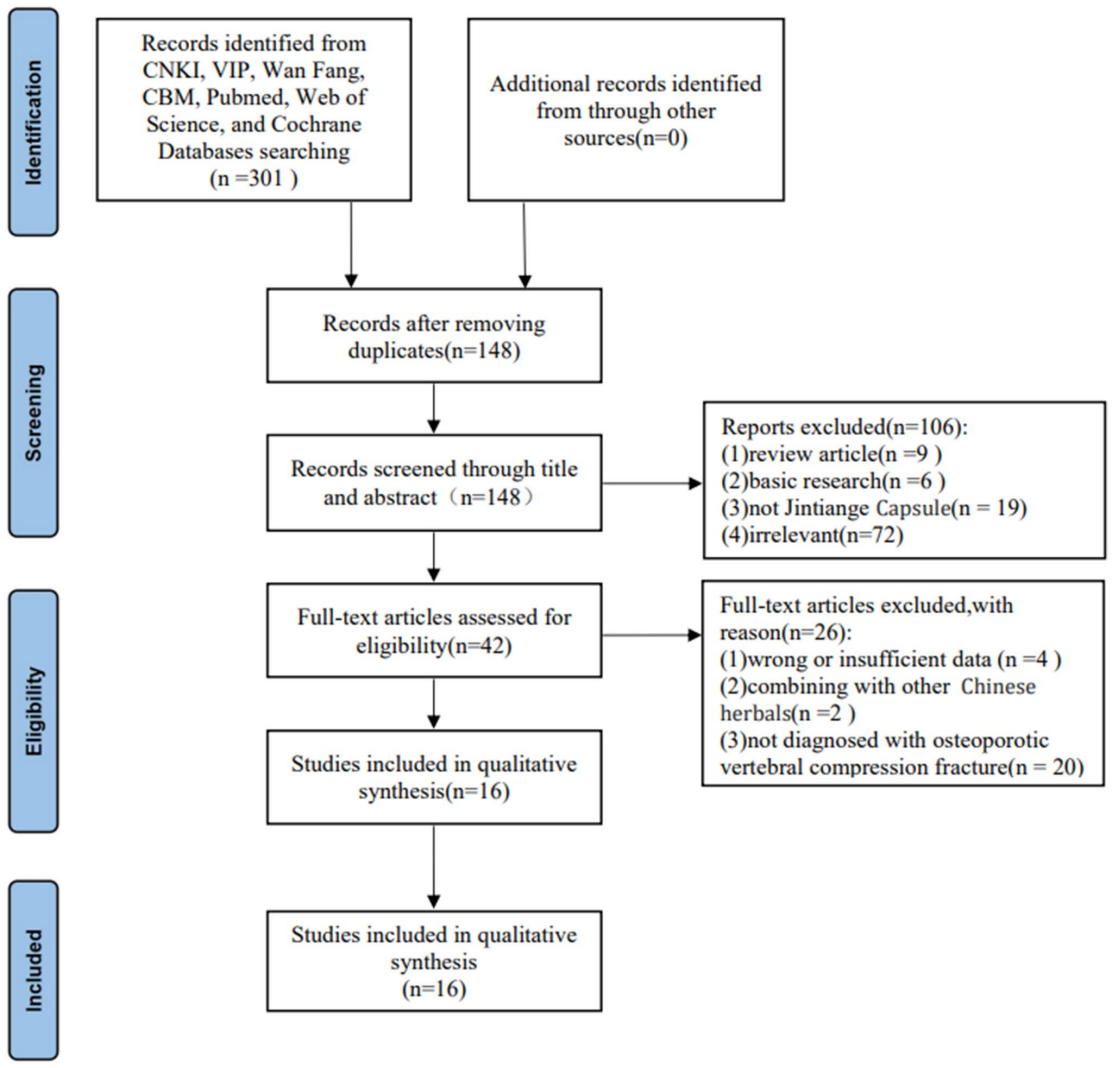
Flowchart of the literature search process.

According to the screening strategy, 148 articles were initially identified after removing duplicate literature. Next, we eliminated 102 articles by scanning the titles and abstracts. After applying the inclusion and exclusion criteria, the full text of the 42 studies met the requirement with the Jintiange Capsule, of which 16 studies on the postoperative treatment of patients with OVCFs ultimately met the inclusion criterias of this meta-analysis (18, 24–38). Figure 1 shows the search process and selection details.

### 3.2 Characteristics of the included studies

Through searching and screening, this meta-analysis included 16 clinical studies, all of which were RCTs on the postoperative treatment of patients with OVCFs with Jintiange capsules. These enrolled studies were all conducted in China. Overall, 16 RCTs included 1,642 participants, of which 822 were in the intervention group and 820 were in the control group. The year of publication of the included trials ranged from 2014 to 2023. All the reported groups in the studies were matched in terms of age, gender, operation, course of disease, and outcome. The operation methods for OVCFs were PVP, PKP, and DHS. Among all studies of the intervention group, 10 studies involved combined therapies and six were single treatment studies with only Jintiange capsules. The daily dose of Jintiange capsules is 1.2 g. The course of interventions was between 1 month and 12 months. Table 1 shows the specific details and characteristics of all the included studies.

**Table 1:** Characteristics of the 16 included studies.

Study	Study design	Sample size (EG/CG)	Operation method	Mean age		Interventions		Course of treatment
				EG	CG	EG	CG	
Xu (2023)	RCT	116 (58/58)	PVP	61.51 ± 5.23	61.54 ± 5.24	Jintiange capsules (1.2 g, tid) + CG	Alendronate (70 mg, qw) + calcium carbonate D3 (600 mg, bid) + salmon calcitonin (20 μg, qd)	3 months
Sun et al. (2023)	RCT	58 (28/30)	PKP	77.64 ± 5.96	76.76 ± 6.47	Jintiange capsules (1.2 g, tid)	Calcium carbonate D3 (600 mg, bid)	3 months
Shu and Zhang (2022)	RCT	106 (53/53)	PKP	67.5 ± 2.7	67.8 ± 2.8	Jintiange capsule (1.2 g, tid)	Conventional western medication	3 months
Qiu et al. (2022)	RCT	150 (75/75)	PVP	65.71 ± 6.85	65.42 ± 7.29	Jintiange capsules (1.2 g, tid) + CG	Calcium carbonate D3 (600 mg, qd) + calcitriol capsule (0.25 μg, qd)	6 months
Yang (2021)	RCT	66 (33/33)	NA	69.32 ± 1.52	69.31 ± 1.55	Jintiange capsules (1.2 g, tid) + CG	Calcium carbonate D3 (750 mg, tid) + alendronate (60 mg, qw)	6 months
Wu et al. (2021)	RCT	78 (39/39)	PKP	66.15 ± 8.12	65.37 ± 8.28	Jintiange capsules (1.2 g, tid) + CG	Calcium carbonate D3 (600 mg, qd) + alendronate (70 mg, qw) + salmon calcitonin (20 μg, qd)	6 months
He et al. (2021)	RCT	86 (43/43)	IFS	66.42 ± 8.82	65.22 ± 6.78	Jintiange capsules (1.2 g, tid)	Calcium carbonate D3 (1,200 mg, bid)	1 month
Han et al. (2021)	RCT	80 (40/40)	PKP	68.8 ± 5.70	70.2 ± 6.2	Jintiange capsules (1.2 g, tid)	Calcium carbonate D3 (600 mg, bid)	6 months
Huang (2020)	RCT	58 (29/29)	NA	70.21 ± 6.58	70.85 ± 6.49	Jintiange capsules (1.2 g, tid)	Calcium carbonate D3 (100 mg, tid) + alendronate (70 mg, qw)	1 month
Huang and Pang (2020)	RCT	128 (64/64)	NA	68.83 ± 2.91	69.02 ± 2.74	Jintiange capsules (1.2 g, tid) + CG	Calcium carbonate D3 (600 mg, bid)	6 months
Wang (2019)	RCT	96 (48/48)	IFS	62.5 ± 8.0	62.30 ± 6.7	Jintiange capsules (1.2 g, tid)	Alfacalcidol (0.5 μg, qd)	6 months
Tan (2019)	RCT	104 (52/52)	PVP	NA	NA	Jintiange capsules (1.2 g, tid)	Calcium (600 mg, qd) + vitamin D3 (125 U, qd)	6 months
Li (2019)	RCT	60 (30/30)	NA	72.5 ± 5.2	73.0 ± 5.3	Jintiange capsules (1.2 g, tid)	Calcium carbonate (750 mg, tid) + alendronate (70 mg, qw)	1 month
Li et al. (2018)	RCT	136 (68/68)	PVP	76.6 ± 8.4	75.9 ± 8.9	Jintiange capsule (1.2 g, tid)	Calcium carbonate D3 + zoledronic acid + calcitriol capsules	3 months
Yan (2015)	RCT	120 (60/60)	PVP	72.5	72.5	Jintiange capsules (1.2 g, tid)	Calcium (200 mg, qd)	3 months
Chen and Bi (2014)	RCT	200 (100/100)	PVP	65.4	65.4	Jintiange capsule (1.2 g, tid) + CG	Calcium carbonate D3 (600 mg, bid) + salmon calcitonin (50 IU, qd)	3 months

RCT, randomized controlled trial; EG, experimental group; CG, control group; PVP, percutaneous vertebroplasty; PKP, percutaneous kyphoplasty; IFS, internal fixation surgery; qd, once a day; bid, twice a day; tid, three times a day; qw, once a week; NA, not available.

### 3.3 Literature quality assessment

**Figure 2:**
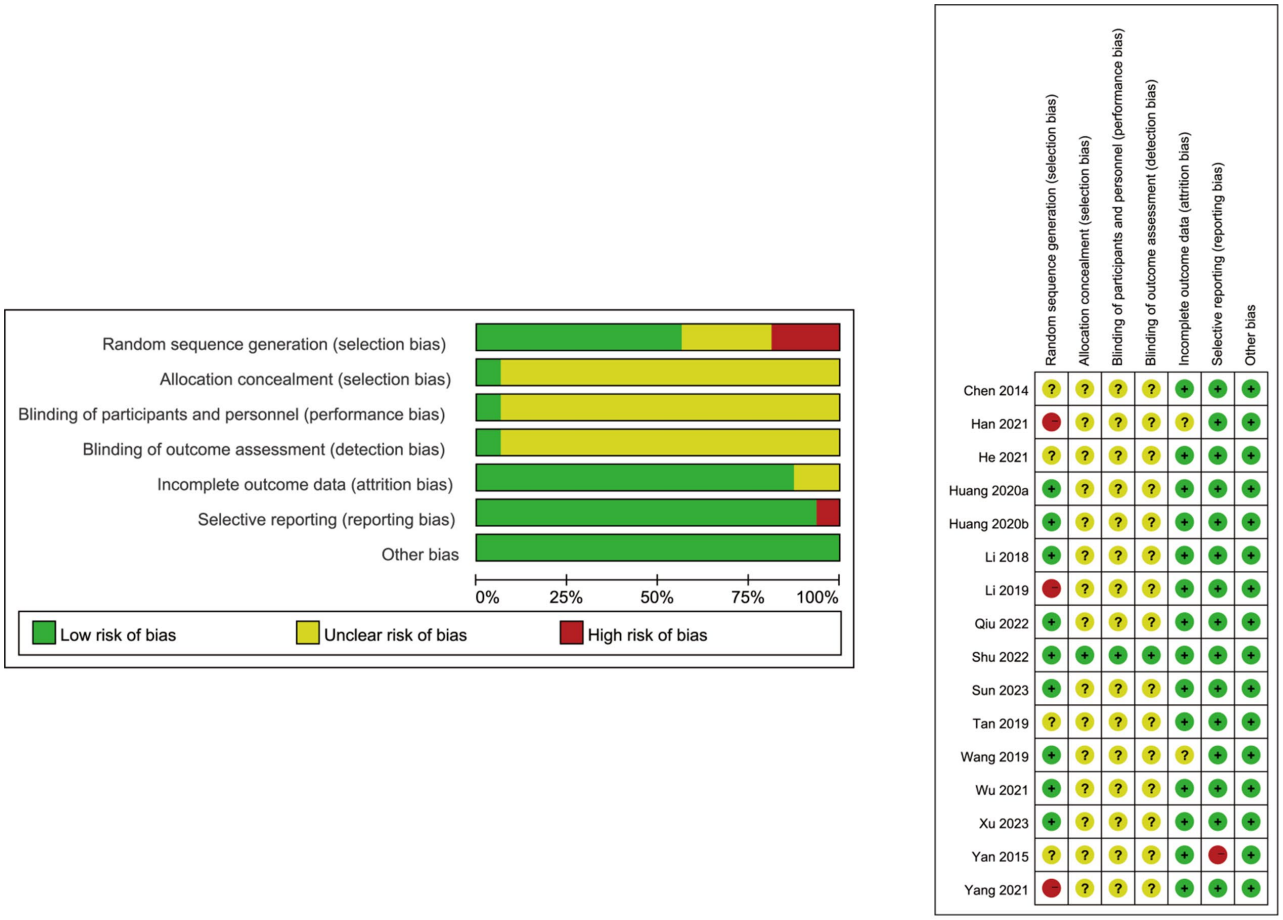
Risk of bias summary of the 16 included studies.

Each RCT employed the Cochrane Collaboration’s tool to evaluate the risk of bias. Most of the 16 articles enrolled in this meta-analysis were randomized controlled trials, among which three studies did not document the random allocation approach (21, 26, 29). Although only one study mentioned the concealment allocation and others did not clearly describe the method (12), it was unlikely to affect the data evaluation. With one of the 16 studies considered to have a low risk of performance bias for the blinding of participants and blinding of outcome assessment (12), the residual studies included remained unclear. Fourteen RCTs did not meet the incomplete outcome data criterion or had no drop-out patient data (12, 18–22, 24, 25, 27–32). One study did not provide a precise explanation of the selective reporting of data (19), which may lead to potential outcomes risk bias. Baseline comparisons were in accordance with the published prespecified analysis plan. No information mentioned that the baseline comparisons were imbalanced, and other biases were not present in the enrolled studies. The details about the risk of bias evaluation for each study are shown in Figure 2.

### 3.4 Results of the meta-analysis

#### 3.4.1 Primary outcomes

##### 3.4.1.1 BMD of the lumbar spine

**Figure 3:**
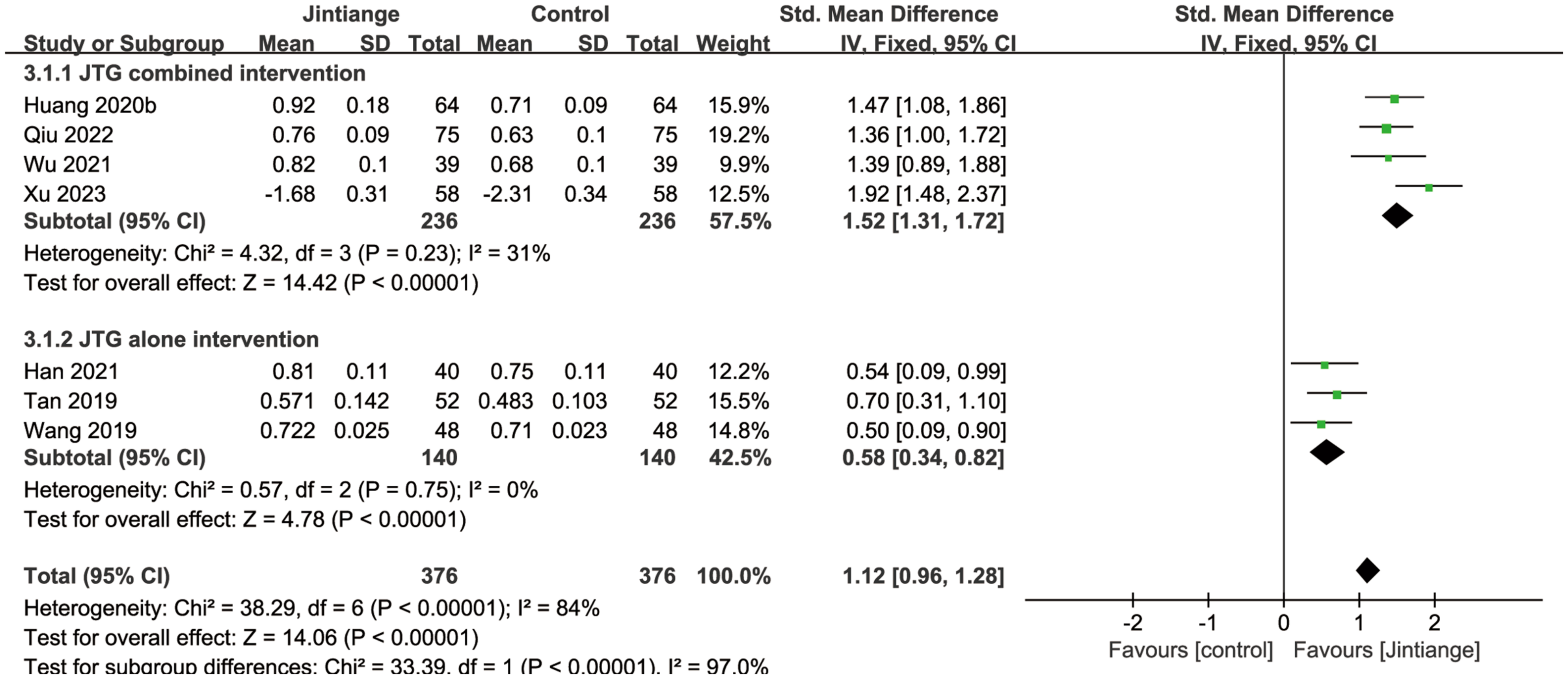
Forest plot of the change in lumbar spine BMD.

Seven studies (28–30, 32, 34, 36, 38) reported BMD changes at the lumbar spine. These enrolled studies showed that the test for the overall effect of the Jintiange capsules significantly increased the BMD of the lumbar spine compared with the controls (SMD = 1.12, 95% CI: 0.96 to 1.28, *p* < 0.00001). Subgroup analyses of the Jintiange capsules combined intervention reported that the *Z* value of the test for overall effect was larger than the Jintiange capsules alone intervention group (*Z* = 14.42), which further suggests that the Jintiange capsules combined intervention appears to be superior at improving the BMD of the lumbar spine (Figure 3).

##### 3.4.1.2 BMD of the femoral neck

**Figure 4:**
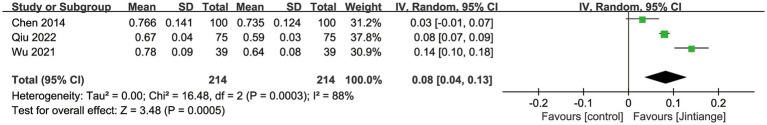
Forest plot of the change in femoral neck BMD.

Three studies reported the effects of the Jintiange capsules on the BMD in the femoral neck (24, 34, 36). The pooled data demonstrated a significant difference in lifting BMD at the femoral neck between the Jintiange capsules and control groups (MD = 0.08, 95% CI: 0.04 to 0.13, *p* = 0.0005) (Figure 4).

##### 3.4.1.3 BMD of the whole body

**Figure 5:**
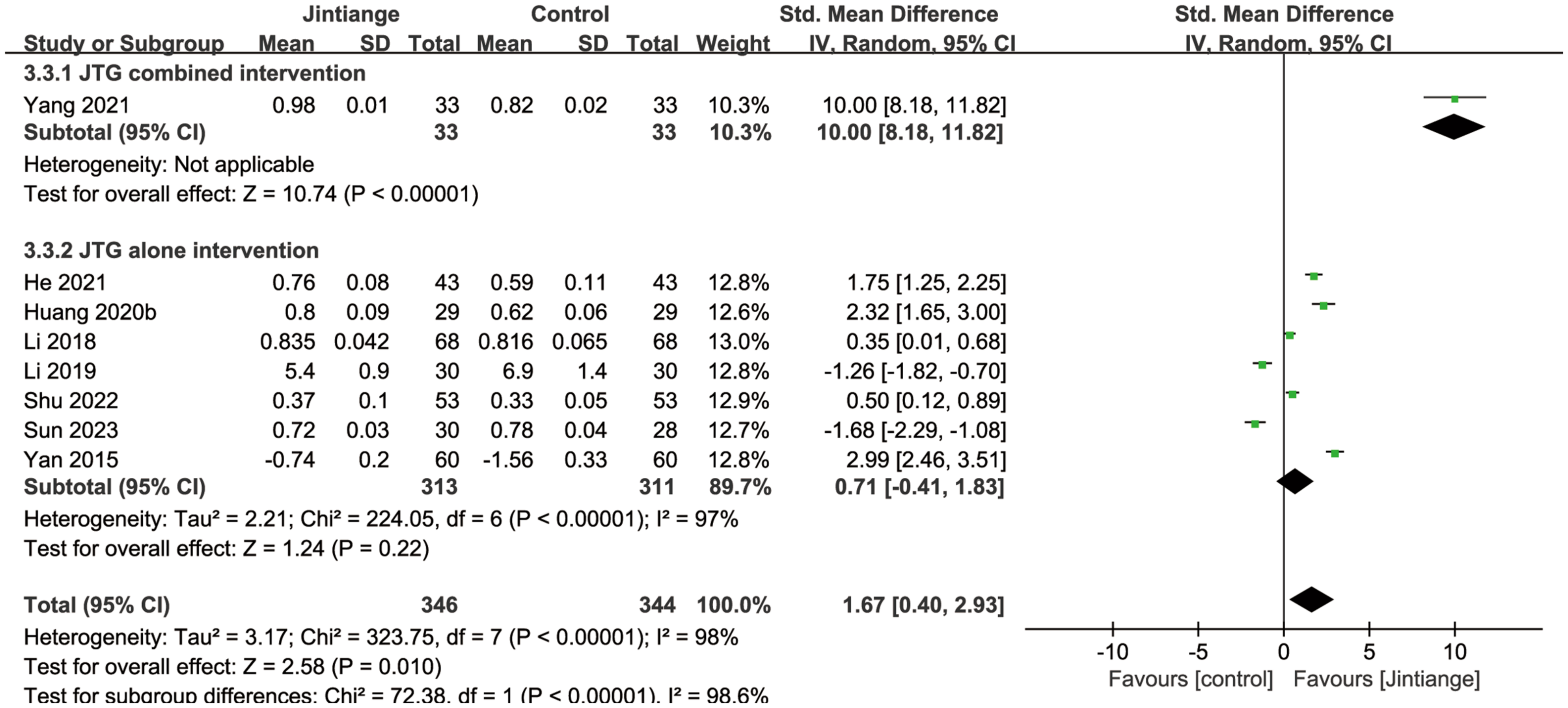
Forest plot of the changes in whole-body BMD.

Eight studies (18, 25–27, 30, 33, 35, 37) reported percentage changes in whole-body BMD. The available data illustrated that compared with the control groups, the overall effect test of the Jintiange capsules showed a significant difference in the BMD of the whole body (SMD = 1.67, 95% CI: 0.40 to 2.93, *p* = 0.01). Additionally, the subgroup analysis showed that the Jintiange capsules combined intervention had a superior effect on whole-body BMD relative to the Jintiange capsules alone (SMD =10.0, 95% CI: 8.18 to 11.82, *p* < 0.00001). However, the pooled results showed no significant difference in whole-body BMD changes between the Jintiange capsules and the control groups (*p* = 0.22) (Figure 5).

##### 3.4.1.4 Effective rate

**Figure 6:**
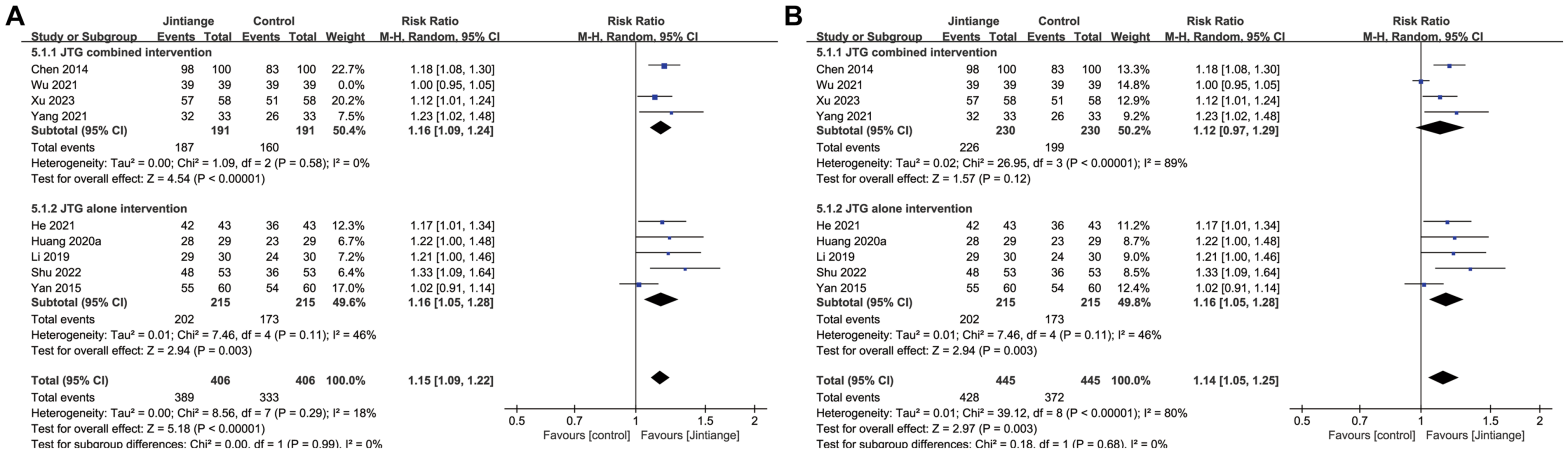
**(A)** Forest plot of the effective rate. **(B)** Forest plot of the effective rate after eliminating the study by Wu.

Nine studies (18, 24, 25, 27, 31, 33–35, 38) showed percentage changes in the effective rate. The test for the overall effect of these nine studies showed that Jintiange capsules significantly improved the effective rate in contrast with control groups (RR = 1.14, 95% CI: 1.05 to 1.25, *p* = 0.003). Subgroup analysis of the effective rate showed that the Jintiange capsules alone intervention was also superior to that of the control groups (*p* = 0.003). However, the pooled results showed that the Jintiange capsules combined intervention did not significantly impact the effective changes between the Jintiange capsules group and the control group (*p* = 0.12). Therefore, the heterogeneity of the Jintiange capsules combined intervention was eliminated after removing Wu et al. The subgroup analysis demonstrated that the *Z* value of the Jintiange capsules combined intervention increased (*Z* = 4.54), the *p*-value decreased (*p* < 0.00001), and the 95% confidence interval was narrowed down (1.09 to 1.24). These changes further suggest that the Jintiange capsules combined intervention appears to be better at increasing the effective rate (Figure 6).

#### 3.4.2 Secondary outcomes

##### 3.4.2.1 VAS

**Figure 7:**
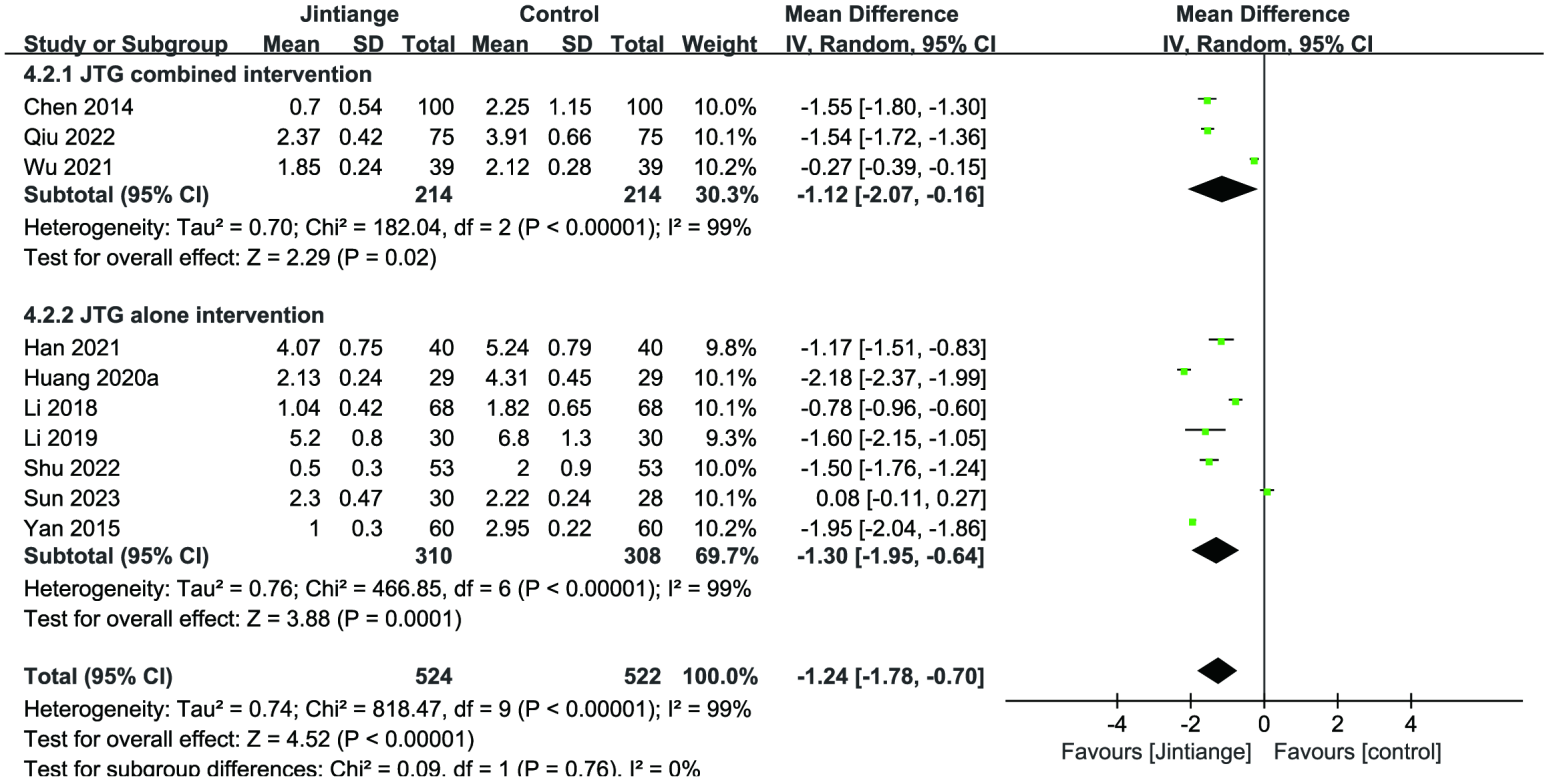
Forest plot of the changes in the VAS.

Ten studies showed the assessment of the VAS. Meta-analysis results showed that the overall effect test reported a significant difference between the Jintiange capsules and the control groups (MD = −1.24, 95% CI: −1.78 to −0.70, *p* < 0.00001) (18, 24–27, 31, 32, 34, 36, 37). Similarly, the subgroup analyses illustrated that both the Jintiange capsules combined and alone interventions remarkably reduced the VAS score relative to controls (*p* = 0.02, *p* = 0.0001) (Figure 7).

##### 3.4.2.2 ODI

**Figure 8:**
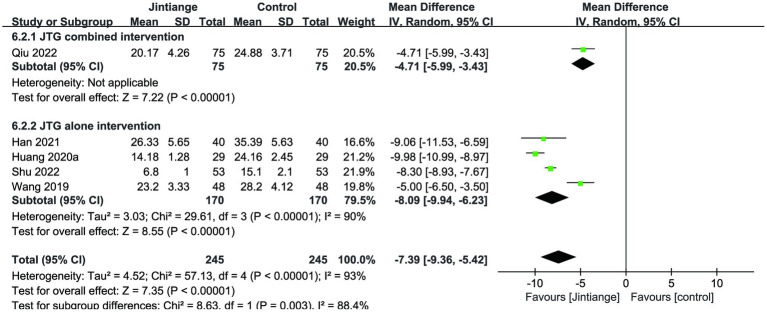
Forest plot of the changes in the ODI.

Five studies (18, 29, 31, 32, 36) reported the percentage changes in the ODI. This meta-analysis illustrated that the overall effect test showed a statistically significant difference in the comparison of ODI levels with the control group (MD = −7.39, 95% CI: −9.36 to −5.42, *p* < 0.00001). The subgroup analysis of the Jintiange capsules combined and alone interventions showed that the Jintiange capsules treatment resulted in a significant decrease in the ODI compared with the control group (*p* < 0.00001) (Figure 8).

##### 3.4.2.3 Cobb’s angle

**Figure 9:**
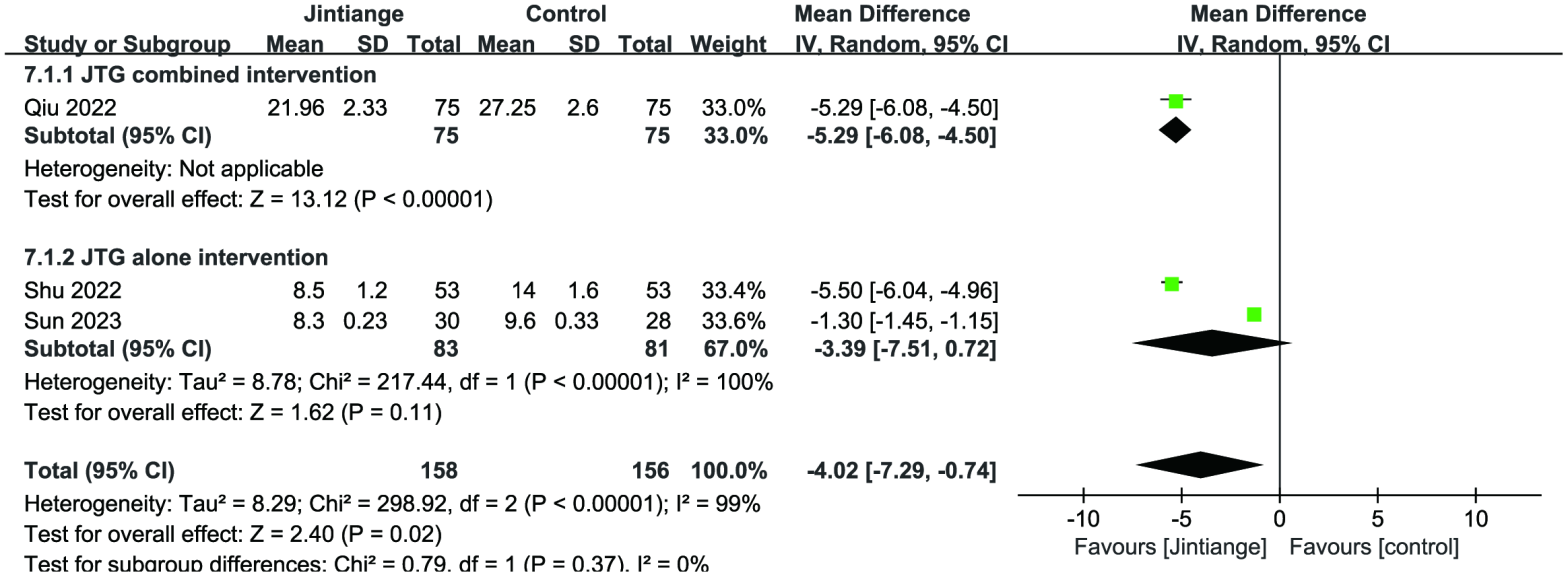
Forest plot of the changes in Cobb’s angle.

This meta-analysis analyzed three studies related to Cobb’s angle (18, 36, 37). The available data of both overall effect test and the subgroup analysis of the Jintiange capsules combined intervention showed that the Jintiange capsules had a superior effect in improving Cobb’s angle than the control group (*p* < 0.00001, *p* = 0.02). However, there was no statistically significant difference between the subgroup of the Jintiange capsules alone and the control condition (*p* = 0.11) (Figure 9).

##### 3.4.2.4 Serum OC

**Figure 10:**
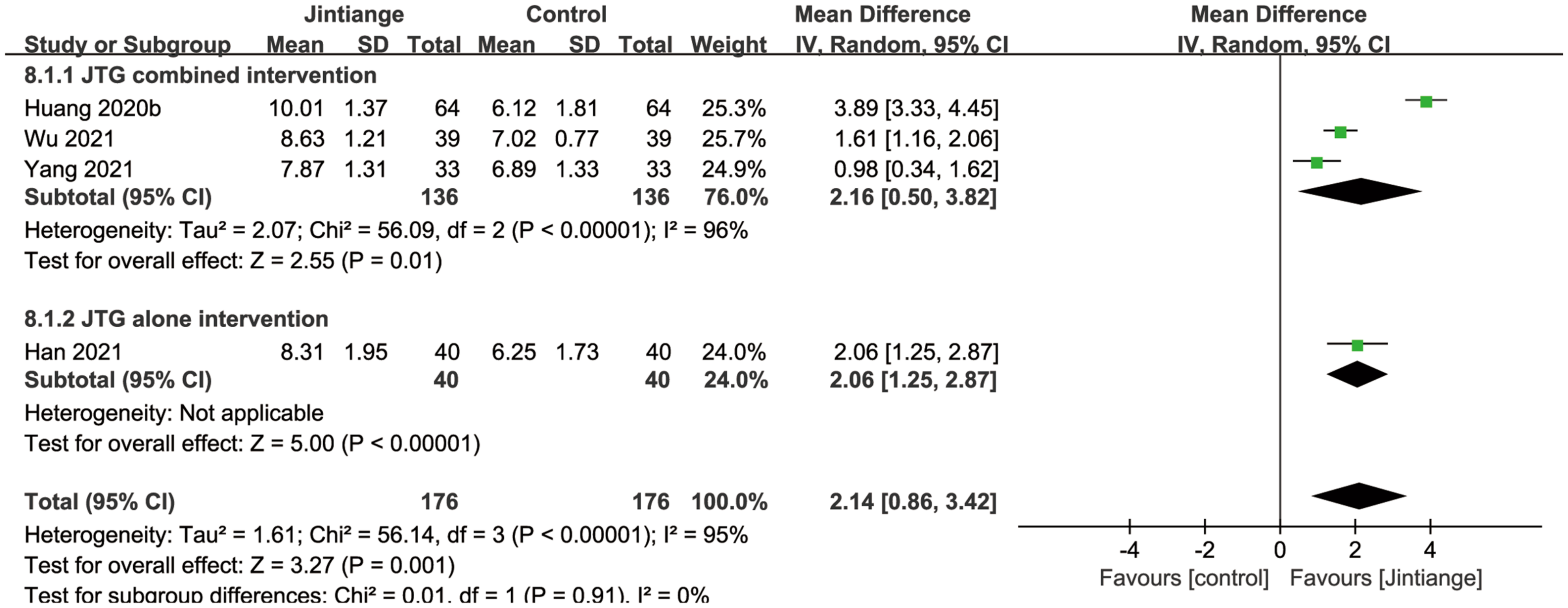
Forest plot of the changes in serum OC.

Four studies (30, 32, 34, 35) reported the changes in the serum OC. As illustrated in Figure 5, the pooled data depicted that the overall effect test showed a significant difference between the Jintiange capsules and control groups (MD =2.14, 95% CI: 0.86 to 3.42, *p* = 0.001). The results of the subgroup analysis indicated that the Jintiange capsules combined and alone interventions also significantly increased serum OC in contrast with the control group (*p* = 0.01, *p* < 0.00001) (Figure 10).

##### 3.4.2.5 Serum ALP

**Figure 11:**
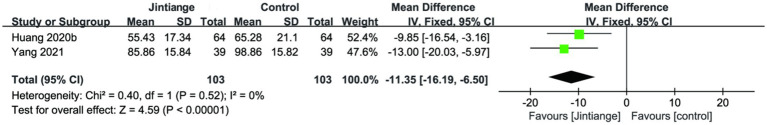
Forest plot of the changes in serum ALP.

Two studies (30, 35) analyzed the changes in serum ALP. The overall effect test of the pooled data demonstrated that the Jintiange capsules significantly reduced serum ALP in contrast with the control group (SMD = −11.35, 95% CI: −16.19 to −6.50, *p* < 0.00001). This result further suggested that the Jintiange capsules combined intervention was remarkable in improving the changes in serum ALP (Figure 11).

#### 3.4.3 Adverse events

Six trials reported information on adverse events and 10 did not (21, 24, 25, 27, 29, 32). The incidence of adverse events was 10/238 in the Jintiange capsules group and 45/238 in the control group. The reporting of adverse events included dry mouth, recurrent fractures, constipation, belching, abdominal distension, diarrhea, nausea, and vomiting. Four studies showed that five patients in the Jintiange capsules group and 28 patients from the control group developed mild gastrointestinal symptoms. In the other two studies, 17 subjects developed recurrent fractures in the controls, among which, one study revealed that five subjects developed recurrent fractures in the Jintiange capsules group. These studies evidenced that the frequency of adverse events was mild, and the treatment was less limited than in the control group, with no severe adverse impacts.

### 3.5 Publication bias and sensitivity analysis

**Figure 12:**
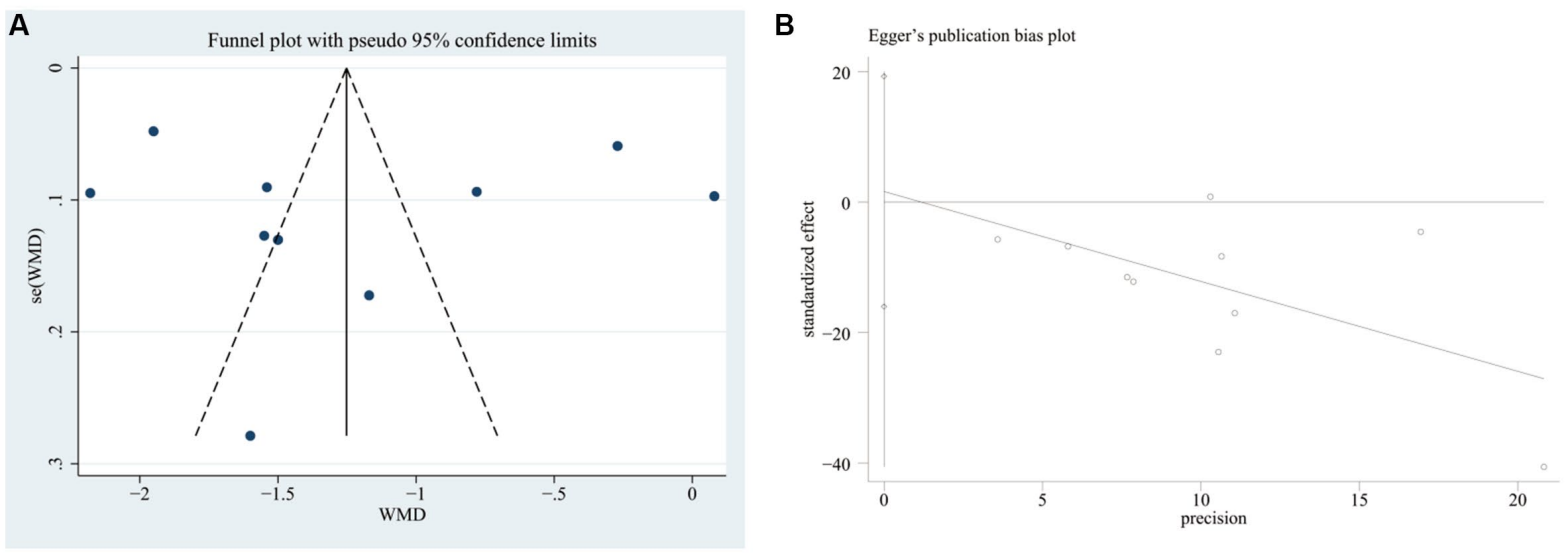
**(A)** Funnel plot of the VAS change. **(B)** Publication bias test for the VAS change.

We plotted the funnel plots along with Egger’s test to evaluate the publication bias of the VAS score in STATA 17 software. The funnel plot showed that these studies were approximately symmetrically distributed on both sides of the regression line. Additionally, the results from Egger’s test showed no significant publication bias for the VAS score (*p* = 0.838) (Figure 12).

**Figure 13:**
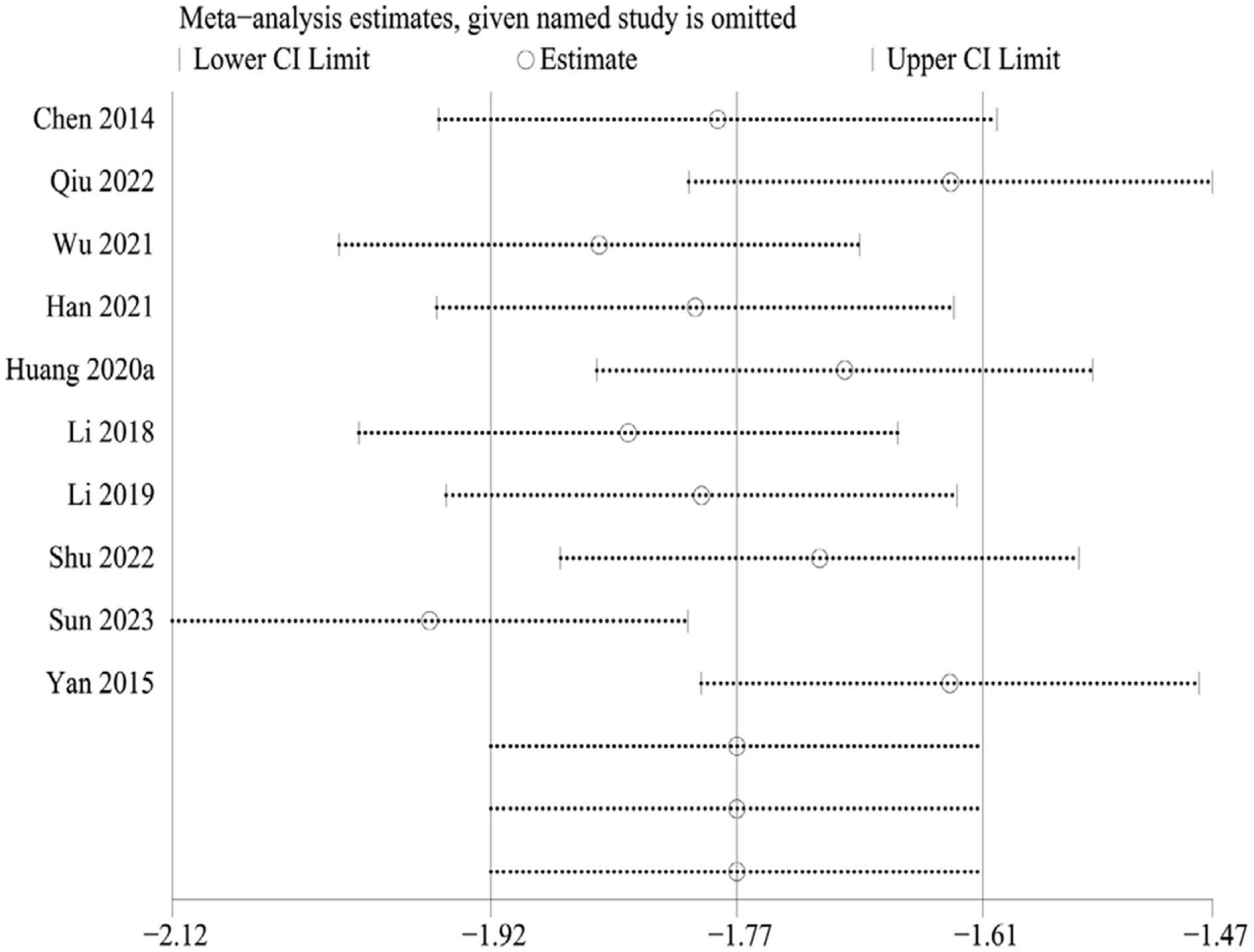
Sensitivity analysis of the VAS change.

We also performed sensitivity analyses for these 10 studies by excluding each article to validate the stability of this meta-analysis, which revealed that there was no statistical effect on the pooled data when any of the articles were eliminated except Sun 2023. However, this study had to be cautiously interpreted when explaining the prognostic results of the VAS due to Sun 2023 appearing to have a potential impact on the subgroup analysis of the Jintiange capsules alone (Figure 13).

## 4 Discussion

OP is a metabolic bone disease characterized by bone density and susceptibility to fracture (39). OVCF is a common secondary disease of OP, with an incidence of 0.307% among people over 50 years old, and is closely associated with low-energy trauma and advancing age. OVCFs may cause severe back pain and kyphosis and increase patient mortality (40). Surgery, such as PVP and PKP, are common alternative therapies for patients who fail to restore the height of the vertebral body through conservative treatments. However, there are inevitable adverse events of cement leakage, pulmonary cement embolism, and new vertebral compression fractures caused by PVP or PKP (41, 42). Similarly, current guidelines suggest that calcium and bisphosphonates can be used as routine oral medications for OP, but these medications may cause adverse events with long-term use, such as renal dysfunction and an increased risk of breast cancer (43). Therefore, effective and safe medications for OVCFs are required to improve this situation. The therapeutic effect of Jintiange capsules on OVCFs has received considerable attention, but research on its anti-OVCF mechanism is limited due to the lack of evidence regarding the effectiveness and safety of Jintiange capsule administration.

Our current study demonstrated that Jintiange capsules exert a prominent anti-OVCF effect. The potent anti-OVCF effect of Jintiange capsules can possibly be attributed to its primary ingredient, artificial tiger bone. Tiger bone has been reported to contain abundant calcium, phosphorus, peptides, and proteins (44). Calcium and phosphorus are fundamental bone minerals involved in many biological processes and can promote the development of bone, maintain the stability of the cytoskeleton, and inhibit osteoclast activity (45). The peptides and proteins can degrade into amino acids *in vivo*, which can potently stimulate increases in intracellular calcium and osteoblast differentiation (46). This meta-analysis indicates that Jintiange capsules maintained and improved the BMD of the lumbar spine, femoral neck, and whole body, and the subgroup analysis of the Jintiange capsules combined intervention showed the same conclusion regarding the lumbar spine and whole-body BMD compared with the control group. After selecting studies with substantial heterogeneity, the overall effect test of subgroup analysis revealed that Jintiange capsules improved the overall effective rate (RR = 1.15, 95% CI: 1.09 to 1.22, *p* < 0.00001, *I*^2^ = 0%). Chronic pain is the major symptom of postoperative treatment for OVCFs (47). In this meta-analysis, the pooled data of the subgroup analysis showed that the Jintiange capsules combined intervention reduced the VAS compared with controls. In addition, Egger’s test revealed no significant publication bias for the VAS. The subgroup analyses of Jintiange capsules also indicated that the Jintiange capsules combined intervention significantly improved ODI and Cobb’s angle compared with the control group. Osteocalcin (OC) is a protein derived from osteoblasts that is vitamin K-dependent and has biological effects on bone metabolism (48). A previous study has shown that Jintiange capsules can prevent bone loss and promote the osteogenic differentiation of BMSCs by regulating the BMP and Wnt-β-catenin pathways in ovariectomized rats, and BMP-2 can increase the expression of osteoblastic markers in cultures of pluripotent cells (49). The result showed that Jintiange capsules significantly raised serum OC levels, and subgroup analyses of the Jintiange capsules alone and combined interventions suggested similar conclusions. Serum ALP is regarded as a diagnostic marker of bone loss in osteoporotic patients and can play an important role in the activation of osteoblasts (50). Empirical evidence in ovariectomized rats has shown that Chinese medicine can significantly increase bone mass and suppress the formation of osteoclasts by inhibiting serum ALP levels (51, 52). The result of this meta-analysis also showed that the Jintiange capsule decreased ALP levels.

Toxicity is the most severe adverse effect of Jintiange capsules. Four studies reported that the adverse effects are relatively mild gastrointestinal symptoms experienced by patients with OVCFs receiving Jintiange capsules, which can be alleviated by stopping treatment. Five participants of the Jintiange capsules group developed recurrent fractures compared with the control group after treatment with Jintiange capsules, which showed a significant reduction in the rate of new vertebral fractures in patients treated with Jintiange capsules. This also showed that Jintiange capsules had a positive effect on reducing severe bone loss in older populations suffering from OVCFs. According to our analysis, the data suggested that Jintiange capsules are safe for the postoperative treatment of OVCFs in the present situation.

Jintiange capsules, the artificial bone tiger powder prepared from several farmed animal skeletons, have similar chemical constituents and pharmacological properties to natural tiger bone, which significantly exhibit anti-inflammatory, analgesic, fracture-healing, and bone metabolism-improving effects that can treat OVCFs (19, 53). An experimental study found that Jintiange capsules can alter the proliferation, differentiation, and mineralization of MC3T3-E1 osteoblasts to increase osteogenesis, which also can inhibit their apoptosis and enhance autophagy by reversing the regulatory effects of the PI3K-AKT and ER stress pathways (54). Another study showed that the main ingredient of tiger bone is calcium phosphate, which can interact with the targets of CALR and CALM1 to promote the synthesis of extracellular phosphate. Therefore, Jintiange capsules can increase bone and cartilage mineralization and regulate multiple signaling pathways (55). Therefore, this meta-analysis showed that Jintiange capsules increased clinical efficacy in improving biochemical markers. Simultaneously, the pooled data showed that Jintiange capsules maintained a relatively high level of serum OC compared with the control treatment, which could be due to the calcium and phosphate in the capsules increasing osteogenesis and the mineralization of osteoblasts, thereby increasing serum OC. Based on the stabilizing regulatory role of Jintiange capsules in bone metabolism, its clinical application deserves further attention in the postoperative treatment of OVCFs.

## 5 Limitations

The 16 included studies had several limitations that should be considered when interpreting the data. First, the sample sizes of the RCTs were too small, and relevant studies may have been overlooked, although we retrieved seven databases without any restriction on language. Second, there were various methodological quality biases because the report on the assessment methods was uneven for the included studies, such as selection bias, performance bias, and reporting bias. Third, the surgical treatment follow-up period ranged from 1 to 12 months, and thorough groupings with regard to more uniform treatment lengths were not put together. Lastly, there is high heterogeneity of the subgroup analysis in the included articles, indicating that diverse conservative Western medication may be utilized in the Jintiange capsules combined intervention group. Therefore, given the limitations of the designs of the studies, future clinical studies targeting the postoperative treatment of OVCFs with Jintiange capsules should avoid the above situations, as the reliability and evidence-based data on the positive effect of Jintiange capsules on the postoperative treatment of OVCFs remains to be improved.

## 6 Conclusion

The data from the studies showed that Jintiange capsules are effective in the postoperative treatment of OVCFs when combined with other therapeutic measures or alone. Their mechanism of action can increase BMD, increase lift effect rates, relieve pain, decrease the ODI, improve Cobb’s angle, raise serum OC levels, inhibit serum ALP levels, and lower the incidence of adverse events, making it a potential medication for treating OVCFs with high safety and effectiveness. Given the limitations of this study, the efficacy of Jintiange capsules for the postoperative treatment of OVCFs will need to be validated in future clinical studies.

## Data availability statement

The original contributions presented in the study are included in the article/Supplementary material, further inquiries can be directed to the corresponding author.

## Author contributions

YF: Conceptualization, Data curation, Formal analysis, Investigation, Methodology, Project administration, Software, Supervision, Visualization, Writing – original draft, Writing – review & editing. WW: Writing – review & editing, Conceptualization, Funding acquisition, Project administration, Supervision, Visualization. MZ: Writing – review & editing, Formal analysis, Validation. JZ: Software, Validation, Writing – review & editing. MT: Validation, Writing – review & editing, Formal analysis.
