# Supplementary material for: Efficacy of the Chinese herbal medicine Jintiange capsules in the postoperative treatment of osteoporotic vertebral compression fractures: a systematic review and meta-analysis
Source: Front Med (Lausanne). 2023 Dec 15;10:1289818. doi: 10.3389/fmed.2023.1289818 (PMC10754969; doi:10.3389/fmed.2023.1289818)
Supplement: Supplementary file 1 [file Table_1.DOCX]

| **NO** | **Databases** | **Search terms** |
| --- | --- | --- |
| 1 | PubMed | (((((((((((((((((((((((Osteoporosis[MeSH Terms])) OR (Osteoporosis, Post Traumatic[Title/Abstract])) OR (Osteoporosis, Post-Traumatic[Title/Abstract])) OR (Post-Traumatic Osteoporoses[Title/Abstract])) OR (Post-Traumatic Osteoporosis[Title/Abstract])) OR (Osteoporosis, Senile[Title/Abstract])) OR (Osteoporoses, Senile[Title/Abstract])) OR (Senile Osteoporoses[Title/Abstract])) OR (Osteoporosis, Involutional[Title/Abstract])) OR (Senile Osteoporosis[Title/Abstract])) OR (Osteoporosis, Age-Related[Title/Abstract])) OR (Osteoporosis, Age Related[Title/Abstract])) OR (Bone Loss, Age-Related[Title/Abstract])) OR (Age-Related Bone Loss[Title/Abstract])) OR (Age-Related Bone Losses[Title/Abstract])) OR (Bone Loss, Age Related[Title/Abstract])) OR (Bone Losses, Age-Related[Title/Abstract])) OR (Age-Related Osteoporosis[Title/Abstract])) OR (Age Related Osteoporosis[Title/Abstract])) OR (Age-Related Osteoporoses[Title/Abstract])) OR (Osteoporoses, Age-Related[Title/Abstract]) AND (((((((Fractures, Compression[MeSH Terms]) OR (Compression Fractures[Title/Abstract])) OR (Compression Fracture[Title/Abstract])) OR (Fracture, Compression[Title/Abstract])) OR (lumbar compression fracture[Title/Abstract])) OR (thoracic vertebral compression fractures[Title/Abstract])) AND ((((jintiange[MeSH Terms]) OR (bionic tiger bone[Title/Abstract])) OR (artificial tiger bone[Title/Abstract])) OR (jintiange capsule[Title/Abstract]) |
| 2 | Web of Science | (((((((((((((((((((((((((((((((TS=(Osteoporosis)) OR TS=(Osteoporoses)) OR TS=(Osteoporosis, Post-Traumatic)) OR TS=(Osteoporosis, Post Traumatic)) OR TS=(Post-Traumatic Osteoporoses)) OR TS=(Post-Traumatic Osteoporosis)) OR TS=(Osteoporosis, Senile)) OR TS=(Osteoporoses, Senile)) OR TS=(Senile Osteoporoses)) OR TS=(Osteoporosis, Involutional)) OR TS=(Senile Osteoporosis)) OR TS=(Osteoporosis, Age-Related)) OR TS=(Osteoporosis, Age Related)) OR TS=(Bone Loss, Age-Related)) OR TS=(Age-Related Bone Loss)) OR TS=(Age-Related Bone Losses)) OR TS=(Bone Loss, Age Related)) OR TS=(Bone Losses, Age-Related)) OR TS=(Age-Related Osteoporosis)) OR TS=(Age Related Osteoporosis)) OR TS=(Age-Related Osteoporoses)) OR TS=(Osteoporoses, Age-Related)) AND TS=(Fractures, Compression)) OR TS=(Compression Fractures)) OR TS=(Compression Fracture)) OR TS=(Fracture, Compression)) OR TS=(lumbar compression fracture)) OR TS=(thoracic vertebral compression fractures)) AND TS=(jintiange)) OR TS=(bionic tiger bone)) OR TS=(artificial tiger bone)) OR TS=(jintiange capsule) |
| 3 | Cochrane Library | #1 MeSH descriptor: [Osteoporosis]  #2 (Osteoporoses or Osteoporosis, Post-Traumatic or Osteoporosis, Post Traumatic or Post-Traumatic Osteoporoses or Post-Traumatic Osteoporosis or Osteoporosis, Senile or Osteoporoses, Senile or Senile Osteoporoses or Osteoporosis, Involutional or Senile Osteoporosis or Osteoporosis, Age-Related or Osteoporosis, Age Related or Bone Loss, Age-Related or Age-Related Bone Loss or Age-Related Bone Losses or Bone Loss, Age Related or Bone Losses, Age-Related or Age-Related Osteoporosis or Age Related Osteoporosis or Age-Related Osteoporoses or Osteoporoses, Age-Related):ti,ab,kw  #3 #1 or #2  #4 MeSH descriptor: [Fractures, Compression]  #5 (Compression Fractures or Compression Fracture or Fracture, Compression or lumbar compression fracture or thoracic vertebral compression fractures):ti,ab,kw  #6 #4 or #5  #7 (jintiange or bionic tiger bone or artificial tiger bone or jintiange capsule):ti,ab,kw  #8 #3 and #6 and #7 |
| 4 | CNKI | (主题:骨质疏松) OR (篇关摘:骨质疏松+骨质疏松症(精确)) AND (主题:椎体压缩性骨折) OR (篇关摘:椎体压缩性折+腰椎椎体压缩性骨折+胸腰椎椎体压缩性骨折+胸腰椎体压缩性骨折+椎体压缩性骨+椎体压缩性骨折(vcfs)((精确)) AND (主题:金天格) OR (篇关摘:金天格胶(精确）） |
| 5 | CBM | #1 "骨质疏松"[不加权:扩展]  #2 "骨质疏松"[常用字段:智能] OR "骨质疏松症"[常用字段:智能]  #3 "骨折, 压缩性"[不加权:扩展]  #4 "压缩性骨折"[常用字段:智能] OR "椎体压缩性骨折"[常用字段:智能] OR "腰椎椎体压缩性骨折"[常用字段:智能] OR "胸腰椎椎体压缩性骨折"[常用字段:智能] OR "脊柱椎体压缩性骨折"[常用字段:智能]  #5 "金天格"[常用字段:智能] OR "金天格胶囊"[常用字段:智能] OR "人工虎骨"[常用字段:智能]  #6 ("骨质疏松"[常用字段:智能] OR "骨质疏松症"[常用字段:智能]) OR ("骨质疏松"[不加权:扩展])  #7 "压缩性骨折"[常用字段:智能] OR "椎体压缩性骨折"[常用字段:智能] OR "腰椎椎体压缩性骨折"[常用字段:智能] OR "胸腰椎椎体压缩性骨折"[常用字段:智能] OR "脊柱椎体压缩性骨折"[常用字段:智能]) OR ("骨折, 压缩性"[不加权:扩展])  #8 (("压缩性骨折"[常用字段:智能] OR "椎体压缩性骨折"[常用字段:智能] OR "腰椎椎体压缩性骨折"[常用字段:智能] OR "胸腰椎椎体压缩性骨折"[常用字段:智能] OR "脊柱椎体压缩性骨折"[常用字段:智能]) OR ("骨折, 压缩性"[不加权:扩展])) AND (("骨质疏松"[常用字段:智能] OR "骨质疏松症"[常用字段:智能]) OR ("骨质疏松"[不加权:扩展])) AND ("金天格"[常用字段:智能] OR "金天格胶囊"[常用字段:智能] OR "人工虎骨"[常用字段:智能]) |
| 6 | Wanfang Database | (主题:(骨质疏松) or 题名或关键词:(骨质疏松) or 题名:(骨质疏松症) and (主题:(压缩性骨折) or 题名或关键词:(椎体压缩性骨折) or 题名或关键词:(腰椎椎体压缩性骨折) or 题名或关键词:(胸腰椎椎体压缩性骨折) or 题名或关键词:(脊柱椎体压缩性骨折)) and (主题:(金天格) or 题名或关键词:(金天格胶囊) or 题名或关键词:(人工虎骨)) |
| 7 | China Science and Technology Journal Database (VIP) | 题名或关键词:(骨质疏松 OR 骨质疏松症) and 题名或关键词:(压缩性骨折 OR 椎体压缩性骨折 OR 腰椎椎体压缩性骨折 OR 胸腰椎椎体压缩性骨折 OR 脊柱椎体压缩性骨折) and 题名或关键词:(金天格 OR 金天格胶囊 OR 人工虎骨) |
